# Characterization of Double-Doped Polymer Optical Fibers as Luminescent Solar Concentrators

**DOI:** 10.3390/polym11071187

**Published:** 2019-07-15

**Authors:** Itxaso Parola, M. Asuncion Illarramendi, Florian Jakobs, Jana Kielhorn, Daniel Zaremba, Hans-Hermann Johannes, Joseba Zubia

**Affiliations:** 1 Department of Applied Physics I, University of the Basque Country (UPV/EHU), Engineering School of Bilbao (EIB), Plaza Ingeniero Torres Quevedo 1, E-48013 Bilbao, Spain; 2 Institut für Hochfrequenztechnik, Technische Universität Braunschweig, 38106 Braunschweig, Germany; 3 Department of Communications Engineering, University of the Basque Country (UPV/EHU), Engineering School of Bilbao (EIB), Plaza Ingeniero Torres Quevedo 1, E-48013 Bilbao, Spain

**Keywords:** polymer optical fibers, PMMA, dopants, luminescent materials, luminescent solar concentrators, green energy

## Abstract

This work reports on a diameter dependence analysis of the performance as luminescent solar concentrators of three self-fabricated polymer optical fibers (POFs) doped with a hybrid combination of dopants. The works carried out include the design and self-fabrication of the different diameter fibers; an experimental analysis of the output power, of the output irradiance and of the fluorescent fiber solar concentrator efficiency; a comparison of the experimental results with a theoretical model; a study of the performance of all the fibers under different simulated lighting conditions; and a calculation of the active fiber length of each of the samples, all of them as a function of the fiber core diameter. To the best of our knowledge, this paper reports the first analysis of the influence of the POF diameter for luminescent solar concentration applications. The results obtained offer a general perspective on the optimal design of solar energy concentrating systems based on doped POFs and pave the way for the implementation of cost-effective solar energy concentrating devices.

## 1. Introduction

Because of the global economic development, the ever-increasing needs and demands of the world population, and environmental concerns, such as global warming, the search for alternative energy sources has become of great urgency. In 2017, 85% of worldwide energy consumption came from fossil fuels, including oil, coal, and natural gas [1]. These kinds of energy production are considered non-renewable, and, eventually, they will become too costly to harvest. Photovoltaic technology (PV) appears to be a promising route to future green energy production, as it has the largest growth prospects among the renewable energy resources. It is abundant, clean and inexhaustible, with high power availability all over the globe. The purpose of converting sunlight into electricity using PV cells has been intensively studied for more than 60 years. Since the beginning, the silicon-based solar cells dominated the field, and significant advances have been achieved since then [2]. However, some drawbacks limit the production of solar energy in a competitive and economically attractive manner. The limited resources of high purity refined silicon, combined with the large areas of solar cells that are required, increase the cost of the system. In addition, a very precise sun tracking and alignment are often needed, which also leads to a rise in the overall cost of the device. It was in the 1970s that the concept of luminescent solar concentrator (LSC) was first proposed by Weber and Lambe [3]. The invention of novel luminescent fluorophores such as quantum dots, semiconductor polymers, and also the Lumogen series of organic fluorophores developed by BASF [4], has renewed the interest in the LSC development in recent years [5,6,7,8,9]. A review covering advancements in LSCs was published in 2012 [10]. In LSCs, luminescent materials are embedded or coated on transparent waveguides so that they absorb part of the sunlight and re-emit and transport it to the edges of the layer where the PV cells or passive optical fibers are attached. These systems present several advantages, such as no need for a sun tracking system, invariable performance under different lighting conditions, and distributed heat dissipation over a large area. Moreover, the use of transparent polymers as the host medium, and the location of the PV cells only at the edges of the active layer, considerably decrease the cost of the PV energy production. However, traditional square LSCs present difficulties in the coupling the output light to PV cells and to passive fibers for light guiding to remote locations. To overcome these limitations, a cylindrical LSC based on doped polymer optical fibers, namely fluorescent fiber solar concentrator (FFSC) appears to be a highly competent solution [11,12,13,14,15]. The structure of POFs adds several benefits, such as easy manipulation by the user, and easy butt-coupling to transparent optical fibers for light waveguiding, which allows spatial separation between the light harvesting system and the final system placement. Moreover, some theoretical studies carried out in the last years suggest that the cylindrical geometry leads to an increase in the concentration factor of the LSC device because of the larger area-ratio between the illuminated length and the edges [16,17,18]. Due to the advantages that FFSCs present, combined with commonly available materials and ease of processing, there exists an opportunity to develop useful and cost-competitive devices that could find a place in niche markets. A potential market lies in building integrated photovoltaics (BIPVs) in cities, where the cost of land for the installation of ground PV is prohibitively high, and the rooftop space is too scarce to accommodate PV modules [10,19,20]. The implementation of LSCs in BIPVs plays a key role in the transition to fully energetically sustainable architecture, so-called net-zero energy buildings (NZEBs), whose energy consumption is nearly fully counterbalanced by renewable energy generated on site, which provides rapid returns on investment. The European commission’s Energy Performance of Buildings Directive requires that all new buildings be NZEB by the end of 2020 [21]. In addition, a further dramatic increase in electrical demand is expected in the near future due to the widespread use of fully electric vehicles. For all these reasons, great efforts are being dedicated to the development of integrated solar-energy technologies.

In this paper, we report, for the first time to our knowledge, a diameter dependence analysis of the FFSC performance of three self-fabricated double-doped POFs of five different diameters. The works carried out comprise the design and fabrication of the fibers, an experimental and theoretical analysis of the output power, of the output irradiance and of the FFSC efficiency, a study of the performance of the fibers under different simulated lighting conditions, and a calculation of the active fiber length of each of the samples. The main objective is focused on providing a complete diameter dependence analysis under AM 1.5G radiation to determine the optimal design characteristics of these novel FFSC systems.

## 2. Materials and Methods

**Figure 1:**
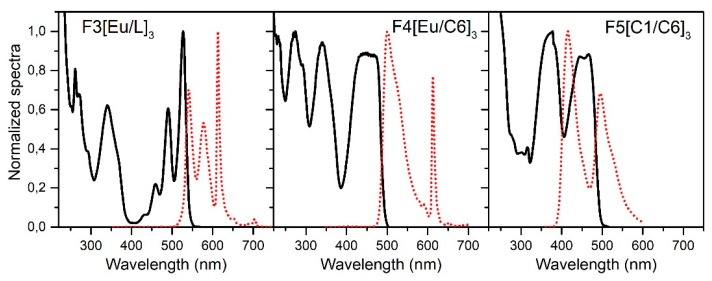
Total absorption and emission bands of the selected hybrid combinations of dopants measured in DCM dissolution. Solid line: absorption. Dotted line: emission.

**Figure 2:**
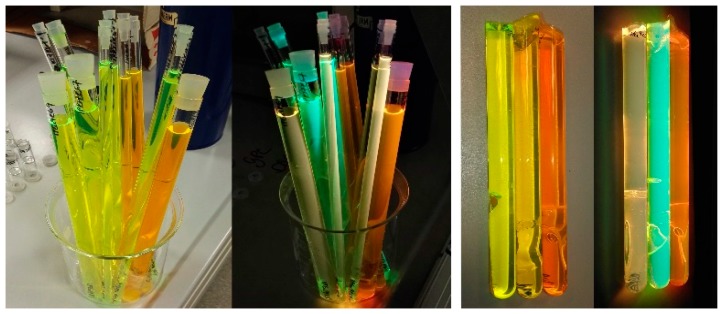
Photographs of the solutions before (**left**) and after (**right**) polymerization under environmental lighting and UV excitation.

The fibers employed in this study were self-fabricated using a double step process of bulk polymerization and fiber drawing, poly(methyl methacrylate) (PMMA) as a host material, and a mixture of dopants including organic dyes (Lumogen Orange/Coumarin-1/Coumarin-6) and a metal-organic material (Eu(TTFA)_3_Phen). The Lumogen Orange (>98%) was obtained from Tokyo Chemical Industry Co., Ltd. (Tokyo, Japan), Coumarin-1 and Coumarin-6 were purchased from Radiant Dyes Laser & Accessoires GmbH (Wermelskirchen, Germany) and sublimated prior use, and Eu(TTFA)_3_Phen was self-synthesized in our laboratory and precipitated in water. The organic dyes are widely known for having broad absorption and emission cross sections, long-term stability, and high quantum efficiencies in PMMA [22,23]. On the other hand, Eu(TTFA)_3_Phen is characterized by its good photo-stability and minimum reabsorption losses. The three selected dopant combinations and concentrations are the following: Eu(TTFA)_3_Phen 0.005 mol % and Lumogen Orange 0.003 mol % (fiber code F3[Eu/L]_3_); Eu(TTFA)_3_Phen 0.005 mol % and Coumarin-6 0.005 mol % (fiber code F4[Eu/C6]_3_); and Coumarin-1 0.007 mol % and Coumarin-6 0.01 mol % (fiber code F5[C1/C6]_3_). For the preform polymerization, a combination of two different dopants, lauroyl-peroxide (Sigma Aldrich, St. Louis, MO, USA) 0.03 mol % (polymerization initiator), and 1-butyl-mercaptan (Acros Organics, Morris Plains, NJ, USA) 0.1 mol % (polymerization inhibitor) were solved in a nitrogen-saturated MMA (>99.8%) (Tokyo Chemical Industry Co., Ltd, Tokyo, Japan) solution at room temperature. The solution was filtered into borosilicate tubes of different diameters (7, 10 and 16 mm) and transferred into a programmable heating cabinet. The preforms were heated up to 100 °C along five days, maintained constant for 1 day, and cooled down to 20 °C along 24 h. Afterward, they were drawn to fiber at 200 °C by applying a constant feed and drawing speed. In a second step, a PC404F-AP (Luvantix, Daejeon, Korea, *n* = 1.404, 589 nm) polymer was applied and cured by UV light irradiation to form the cladding layer. All the fibers are step-index. For each of the dopant combinations, five different fiber diameters were fabricated, which are summarized in Table 1. The diameter tolerance is less than 5% in all cases. Their total absorption and emission bands are shown in Figure 1, and as can be seen, in all cases the emission is red-shifted in comparison to the absorption. This means our fibers absorb the sunlight in regions where the silicon photovoltaic cells have a worse response (closer to the blue and near UV region) and re-emit it in longer wavelengths where the silicon cells are more efficient. However, when using organic dyes as dopant materials, there exists an overlap between the absorption and emission bands that can cause reabsorption events, and, consequently, a possible increase in the optical losses [24,25,26]. Photographs of the solutions before and after polymerization under environmental lighting and UV excitation are shown in Figure 2. Further information about the dopant selection and the polymer analysis can be found in [27].

**Figure 3:**
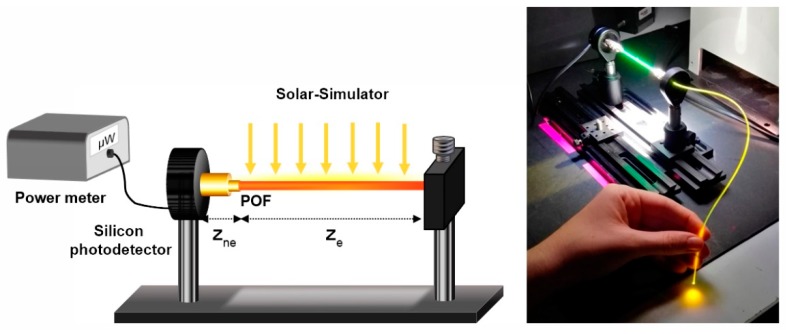
Schematic of the experimental set-up employed for the characterization under solar simulator (**left**). z*_e_* represents the illuminated fiber length, and z*_ne_* is the non-excited fiber length up to the detector; photograph of one of the fibers in the set-up under solar simulator excitation (**right**).

The characterization of the fibers as FFSCs was carried out employing a solar-simulator (Newport Oriel Desktop 91160-1000, Irvine, CA, USA) under the standard 1 Sun AM 1.5G solar radiation (1000 W/m^2^), which is always calibrated prior use [28]. The fibers were placed perpendicularly to the calibrated light source at 24 cm, allowing uniform side excitation in an area of 6 × 6 cm^2^. The acquisition of the emission spectra was realized using a fiber-optic spectrometer (Ocean Optics USB2000, Dunedin, FL, USA). For measuring the output power at one of the fibers ends, a silicon photodetector (Thorlabs S120VC Newton, NJ, USA) and a double-channel power meter (Thorlabs PM320E) were employed. For the study of the performance under different lighting conditions, three optical density (OD) filters (1, 0.5 and 0.2) were employed to attenuate the intensity of the solar-simulator, and the illuminated fiber length z*_e_* was adjusted to 4.5 cm due to the filter characteristics. A schematic representation of the experimental set-up and a photograph of one of the samples under the solar simulator are shown in Figure 3.

## 3. Results and Discussion

### 3.1. Output Power, Output Irradiance, and FFSC Efficiency

The output power of each of the samples, *P*_out_, was measured under AM 1.5G excitation employing an illuminated fiber length of 6 cm and a non-excited fiber length of 3.3 cm (SMA connector). The optical losses in the non-exited length are small in comparison to the high efficiency of the fiber, in the order of 0.05 cm^−1^ in similar doped fibers [24]. From these power values, two parameters were calculated: the output irradiance, *I*_out_, and the FFSC efficiency, η*_FFSC_*. The first was calculated from the *P*_out_ and the fiber end-surface as *I*_out_ = *P*_out_/πr^2^, with r being the fiber core radius. On the other hand, the η*_FFSC_* was estimated as the ratio between the output power at one of the fiber ends, and the excitation-lamp power, *P*_lamp_, as $\eta_{FFSC}\left(\%\right)\;=\;100\left(P_{\mathrm{out}}{/P}_{\mathrm{lamp}}\right)$ It has to be noted that the value of *P*_lamp_ varies for each of the analyzed fiber diameters since it is calculated taking into account the illuminated fiber surface perpendicular to the vertical direction of the flow, as $P_{\mathrm{lamp}}{\;=\;\mathrm{Iz}}_{e}2r$, where I = 1 mW/mm^2^ is the excitation irradiance.

**Figure 4:**
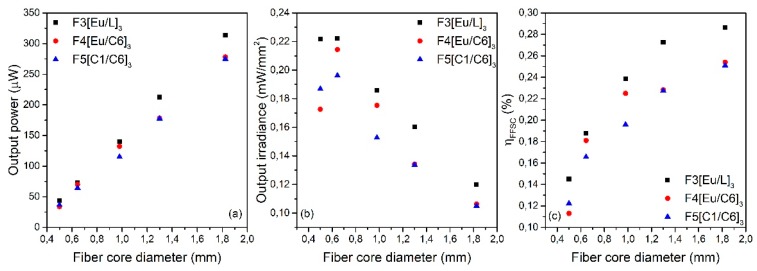
Output power (**a**); output irradiance (**b**); and *η_FFSC_* (**c**) for the three double-doped samples as functions of the fiber core diameter.

Figure 4 illustrates the experimentally measured output power (Figure 4a), output irradiance (Figure 4b), and η*_FFSC_* (%) (Figure 4c) for the three double-doped samples. The data were plotted as functions of the fiber core diameter, as it corresponds to the active volume of the fibers. Firstly, focusing on Figure 4a, it can be seen that the output power shows, in all cases, a nearly linear dependence with the fiber core diameter. Therefore, the greatest power values are obtained for the case of the thickest fiber core diameter since there is greater light–material interaction. Secondly, Figure 4b illustrates the irradiance at one of the fiber ends, showing greater concentration irradiances for smaller core diameters. Finally, from the graph (Figure 4c), it can be seen that the FFSC efficiency increases with the fiber core diameter, but it shows a tendency to saturation. The greatest value is obtained for the thickest sample of F3[Eu/L]_3_, namely 0.29%. The values of the experimental data of Figure 4 are gathered in Table 2. This table also collects the experimental values obtained when using a reflective layer below the fiber at a distance of 4.5 cm. As can be seen, in all cases, the obtained results improve significantly, as this reflective layer bounces back to the fiber the light that was not absorbed directly from the light source. A remarkable performance is obtained for the F3[Eu/L]_3_ 2 mm fiber, with an output power value of 540 μW and a η*_FFSC_* of 0.5% for just one piece of fiber of 6 cm long.

**Figure 5:**
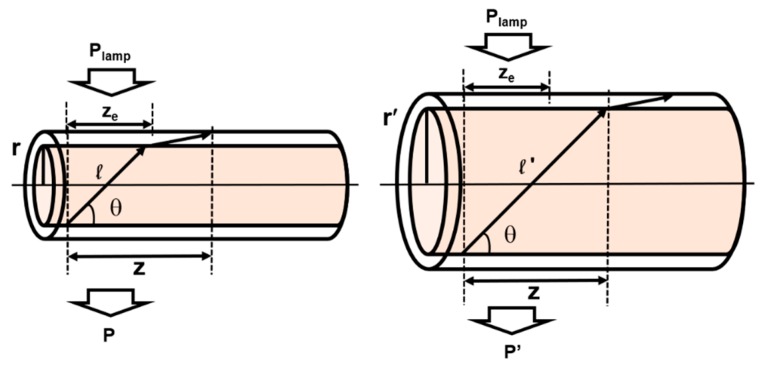
A representative model of the transversal excitation conditions and of the distance covered by rays reflecting at the cladding-air interface in fibers with the same cladding and different core radius (r < r′). *P* and *P′* represent the power of the transversal excitation light (*P*_lamp_) that is not absorbed by the doped core and goes through the fiber. Fibers with greater core radius absorb more light transversally than fibers with smaller core radius (*P* > *P′*).

**Figure 6:**
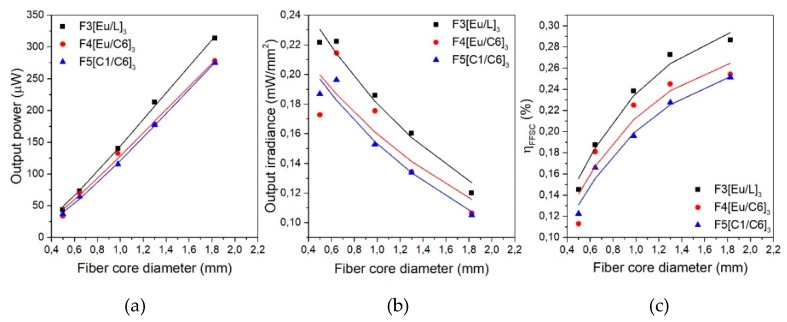
Output power (**a**); output irradiance (**b**), and η*_FFSC_* (**c**) for the three double-doped samples as functions of the fiber core diameter. Symbols: experimental data; solid lines: fittings of Equation (2). The relative errors for the output power, output irradiance and η*_FFSC_* are 4%, 3% and 1%, respectively.

The generation and propagation of light in doped POFs can be described by the so-called rate equation [29,30,31]. The model consists of two coupled differential equations, where one equation gives the variation of the excited state population with time, and, the other, the evolution of the emitted power with the fiber length. By working out these equations, the output power in steady-state and fluorescence regime (no amplification occurring inside the fiber) can be described with the following equation:

(1) $$P_{\mathrm{out}}\;=\;I\;\left(\frac{\lambda_{p}}{\lambda }\right)\left(\frac{\sigma_{sp}^{2}\left(\lambda \right)}{\alpha \left(\lambda \right)}\right)\beta 2r\left({1-e}^{-\alpha \left(\lambda_{p}\right)2r}\right)\left({1-e}^{-\alpha \left(\lambda \right)z_{e}}\right)$$

where I is the excitation irradiance; α(λ*_p_*) represents the absorption coefficient at absorption wavelengths λ*_p_*; α(λ) is the total attenuation coefficient at emission wavelengths λ (mainly due to reabsorption processes); σ*_sp_*(λ) denotes the probability for spontaneous emission to take place at the emission wavelength λ; β is the fraction of the randomly emitted light that is guided and propagated through the fiber [32]; and z*_e_* the excitation fiber length. The term $\left({1-e}^{-\alpha \left(\lambda_{p}\right)2r}\right)$ represents the fraction of power absorbed by the fiber under transversal excitation (see Figure 5). Using Equation (1), if the excitation irradiance and the z*_e_* are maintained constant, as it occurs in our experimental measurements, the dependence of *P*_out_, *I*_out_, and η*_FFSC_* with the fiber radius will be written as:

(2) $$\begin{array}{c} P_{\mathrm{out}}\left(r\right){\;=\;C}_{1}r\left({1-e}^{-\alpha \left(\lambda_{p}\right)2r}\right),\\ I_{\mathrm{out}}\left(r\right)\;=\;\frac{C_{2}}{r}\left({1-e}^{-\alpha \left(\lambda_{p}\right)2r}\right),\;\\ \eta_{FFSC}\left(r\right){\;=\;C}_{3}\left({1-e}^{-\alpha \left(\lambda_{p}\right)2r}\right) \end{array}$$

where C_1_, C_2,_ and C_3_ represent constant magnitudes. The results of the fitting of Equation (2) to the experimental points shown in Figure 4 are illustrated in Figure 6. As can be seen, the experimental results substantially agree with the dependence given by Equation (2). It should be noted that the values of α(λ_p_) obtained from the fittings would correspond to all the absorption wavelengths incident on the fiber, and are 1.32 ± 0.20 mm^−1^, 1.31 ± 0.30 mm^−1^, and 1.24 ± 0.06 mm^−1^ for F3[Eu/L]_3_, F4[Eu/C6]_3_, and F5[C1/C6]_3_, respectively. Even so, other physical effects should be taken into account in the rate equations in order to explain more precisely our experimental results. One of these effects is the higher absorption losses that occur when the core radius is larger, due to the longer path covered by rays reflecting at the cladding-air interface inside the doped core as compared to the path inside the undoped cladding. A simple representation of this can be seen in Figure 5. Another possible effect would be related to the fact that, in fibers with smaller diameters more internal reflections occur along the same fiber length, which would also increase the probability of losses.

### 3.2. Performance under Different Lighting Conditions

**Figure 7:**
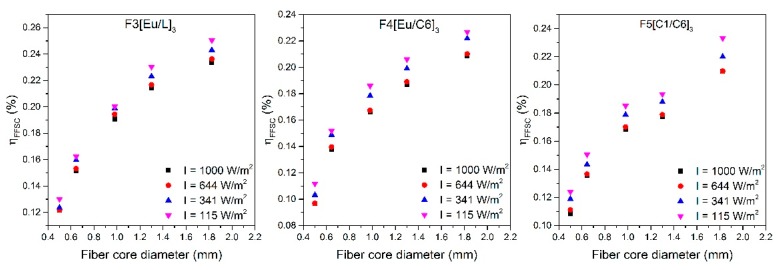
Evolution of the η*_FFSC_* of the three double-doped combinations for different weather scenarios as a function of the fiber core diameter. The illuminated fiber length is 4.5 cm in all cases.

In this section, the performance of the different diameter fibers under several weather scenarios is studied. To simulate these scenarios, OD filters were employed to attenuate the power of the incident radiation [27]. All of the simulated weather conditions were tested for five different fiber diameters of each three dopant combinations. Figure 7 shows the calculated η_FFSC_ values as a function of the fiber core diameter for the four simulated weather cases. From these graphs, it can be seen that the efficiency pattern remains invariable for all simulated weather conditions, i.e., the efficiency increases with the fiber diameter, but it tends to saturate asymptotically following the same trend for all excitation irradiances. In addition, slightly higher efficiency values are obtained as the pump irradiance is decreased for all the fiber diameters, as photodegradation of the dopant molecules increases with greater pump irradiances [27]. It has to be noted that all fiber diameters undergo similar efficiency improvements of around 10%, 7%, and 1% for the cases of cloudy winter day (OD 1), cloudy summer day (OD 0.5), and scattered clouds (OD 0.2), respectively. These values are valid for the three dopant combinations. As a conclusion, it was successfully demonstrated that all the samples presented invariable performance under different weather scenarios, even showing small η*_FFSC_* improvement for cloudy-day conditions.

### 3.3. Power-Saturation Fiber Length

**Figure 8:**
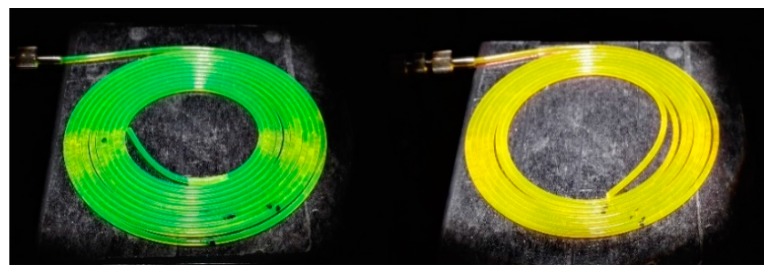
Photograph of two of the samples rolled into several loops under the solar simulator excitation.

The evolution of the output power as a function of the illuminated fiber length was studied for a pump irradiance of 1000 W/m^2^. For this purpose, the fibers were rolled gradually into loops marking the length of each measurement (see Figure 8) and the output power was measured stepwise.

**Figure 9:**
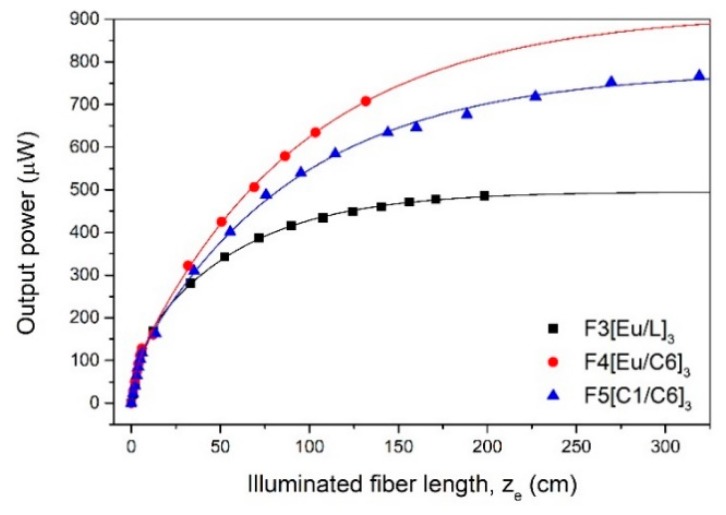
Evolution of the output power as a function of the illuminated fiber length for 1 mm diameter samples. Symbols: experimental points; solid lines: fitted curves.

The output power measured in one of the fiber ends tends to saturate for a certain fiber length due to several loss sources along the light propagation path. This power saturation can be derived from Equation (1) as $P_{\mathrm{sat}}\left(r\right){\;=\;C}_{4}r\left({1-e}^{-\alpha \left(\lambda_{p}\right)2r}\right)$, where C_4_ is a constant magnitude. The power-saturation fiber length *L*_sat_ is given by the term 1/α(λ). From the *P*_sat_, the saturation irradiance can be calculated as $I_{\mathrm{sat}}{\;=\;P}_{\mathrm{sat}}{/\pi r}^{2}$. We followed the criteria of defining *L*_sat_ as the length at which the variation of the output power is less than 0.5% [27]. Taking into account the uncertainty of the detector, the relative error in the calculation of *L*_sat_ is less than 20% in all cases. An example of the experimental data is illustrated in Figure 9, corresponding to the 1 mm diameter samples. The values of *P*_sat_, *L_sat_*, and *I*_sat_ of all fibers analyzed are gathered in Table 3. As can be seen, *P*_sat_ increases with the fiber radius as it was expected. However, *L*_sat_ reaches its maximum at around 1 mm of fiber diameter. This effect would be explained by taking into account a greater contribution of the losses associated with the internal reflections as the fiber radius decreases at small fiber diameters and an increase of the absorption losses while the fiber radius increases.

From the results shown in Table 3, it can be seen that that F4[Eu/C6]_3_ and F5[C1/C6]_3_ combinations show better performance with distance than F3[Eu/L]_3_ in terms of *I*_sat_. An interesting result to be noted is that, with the combination of Coumarin-1 and Coumarin-6, the irradiances obtained for the three smallest diameters are greater than the intensity of the sunlight in the earth surface, which is estimated to be around 1 mW/mm^2^. This means, that a single 2.35 m long fiber, with a core diameter of 0.5 cm, is able to concentrate a sunlight equivalent irradiance. These results may be of great interest in the design and implementation of FFSCs.

## 4. Conclusions

In this work, a self-fabrication and analysis of the diameter dependence of the FFSC performance of three different double-doped POFs were reported for the first time to our knowledge. The evolution of the output power, of the output irradiance and of the η*_FFSC_* with the fiber core diameter were experimentally and theoretically analyzed. It was seen that the output power increases almost linearly with the fiber core diameter, whilst greater output irradiances are obtained for smaller core diameters. The FFSC efficiency increases with the fiber core diameter, but it shows a tendency to saturation. These results may be due to limited capacity to absorb the incident power. Moreover, it was demonstrated that all fiber diameters show invariable performance under different lighting conditions, even observing some slight improvements for lower excitation irradiances. These improvements were calculated to be of around 10%, 7%, and 1% for the cases of a cloudy winter day, cloudy summer day, and scattered clouds, respectively, for all fiber diameters. Finally, the saturation fiber length was studied, yielding highly promising results for FFCS systems. As an example, with F5[C1/C6]_3_ fibers, irradiances comparable to those that would impinge directly from the sun are obtained for the three smallest diameters.

As an overall conclusion from this work, when trying to find the optimum fiber diameter for a specific application, the desired characteristics of the device should be taken into account, and a compromise between the output power and the output irradiance should be taken. For applications where the power represents the key parameter, such as in indoor illumination systems, greater fiber core diameters should be used, whereas, if the output irradiance is the desired parameter, like in solar-to-electricity conversion with PV cells, smaller diameters would be more efficient. These results offer a general perceptive to the user to facilitate the optimal design of the FFSC system for a specific target application and contribute to the promising development of this technology in fields such as BIPV, small-scale clean energy production, and indoor lighting.

## Figures and Tables

**Table 1:** Summary of the five fiber diameters.

Total Fiber Diameter (μm)	Active Core Diameter (μm)	Cladding Thickness (μm)
600	500	50
750	645	47
1000	980	10
1500	1300	100
2000	1825	87

**Table 2:** Output power in (μW), output irradiance in (mW/mm^2^) and η*_FFSC_* in (%) for the three samples and different fiber diameters without and with a reflective layer at 4.5 cm from the fiber.

	Diameter (mm)	F3[Eu/L]_3_			F4[Eu/C6]_3_			F3[C1/C6]_3_		
		*P*_out_	η*_FFSC_*	*I*_out_	*P*_out_	η*_FFSC_*	*I*_out_	*P*_out_	η*_FFSC_*	*I*_out_
**Without Reflective Layer**	0.6	44	0.15	0.22	34	0.11	0.17	37	0.12	0.19
	0.75	73	0.19	0.22	70	0.18	0.21	64	0.17	0.20
	1.0	140	0.24	0.19	132	0.22	0.18	115	0.2	0.15
	1.5	213	0.21	0.16	178	0.23	0.13	177	0.23	0.13
	2.0	314	0.29	0.12	278	0.25	0.11	275	0.25	0.11
**With Reflective Layer**	0.6	87	0.29	0.44	66	0.22	0.34	72	0.24	0.37
	0.75	135	0.35	0.42	133	0.34	0.41	124	0.32	0.38
	1.0	265	0.46	0.36	238	0.40	0.32	217	0.37	0.29
	1.5	374	0.48	0.28	320	0.41	0.24	308	0.40	0.24
	2.0	540	0.50	0.21	462	0.42	0.18	465	0.43	0.18

**Table 3:** Saturation fiber length *L*_sat_ (cm), saturation output power *P*_sat_ (μW), and saturation irradiance *I*_sat_ (mW/mm^2^) for five different fiber diameters. The relative error in *L*_sat_ is less than 20% in all cases.

Fiber Ø (mm)	F3[Eu/L]_3_			F4[Eu/C6]_3_			F5[C1/C6]_3_		
	*L*_sat_	*P*_sat_	*I*_sat_	*L*_sat_	*P*_sat_	*I*_sat_	*L*_sat_	*P*_sat_	*I*_sat_
0.6	138	140	0.71	288	158	0.80	235	203	1.04
0.75	203	256	0.78	458	380	1.16	496	430	1.32
1.0	240	563	0.75	647	1005	1.33	624	890	1.18
1.5	173	615	0.66	308	876	0.66	203	895	0.67
2.0	301	1041	0.40	280	935	0.36	213	1022	0.39
